# Regulation of Erythropoietin Activity in Clear Renal Cell Carcinoma

**DOI:** 10.3390/ijms26083777

**Published:** 2025-04-17

**Authors:** Bojana B. Beleslin Čokić, Sandra Bižić Radulović, Tijana Subotički, Vladan P. Čokić, Constance T. Noguchi, Nebojša Bojanić, Svetozar Damjanović

**Affiliations:** 1 Department for Sero and Molecular Diagnostics, Institute of Virology, Vaccines and Sera “Torlak”, Vojvode Stepe 458, 11221 Belgrade, Serbia; 2 Clinic of Hematology, University Clinical Center of Serbia, Dr Koste Todorovića, 11000 Belgrade, Serbia; bizics@yahoo.com; 3 Department of Molecular Oncology, Institute for Medical Research, National Institute of Republic of Serbia, University of Belgrade, Dr Subotića 4, 11129 Belgrade, Serbia; tijana@imi.bg.ac.rs (T.S.); vl@imi.bg.ac.rs (V.P.Č.); 4 Molecular Medicine Branch, National Institute of Diabetes and Digestive and Kidney Diseases, National Institutes of Health, Bethesda, MD 20892, USA; connien@niddk.nih.gov; 5 Clinic for Urology, University Clinical Center of Serbia, Resavska 51, 11000 Belgrade, Serbia; bojanicnebojsa@gmail.com; 6 Clinic of Endocrinology, University Clinical Center of Serbia, Dr Subotića 13, 11000 Belgrade, Serbia; svetadamjanovic@gmail.com

**Keywords:** clear renal cell carcinoma, von Hippel–Lindau gene, erythropoietin, hypoxia-inducible factor-1, JAK2/STAT5A pathway

## Abstract

Clear-cell renal cell carcinoma (ccRCC) is associated with the mutated von Hippel–Lindau (VHL) gene leading to the activation of hypoxia-inducible factor 1A (HIF1A) and subsequent overexpression of erythropoietin (EPO). We analyzed tumor and healthy tissues from 43 ccRCC patients after radical nephrectomy and cultured 786-O (biallelic *VHL* inactivation) and Caki-1 (wild-type *VHL*) cells in normal (21% O_2_) and low oxygen (3% O_2_) with 10% and 2% fetal bovine serum (FBS). DNA sequencing, including Sanger sequencing, MLPA and LOH, revealed 27 somatic mutations of *VHL* in ccRCC. HIF1A protein showed decreased or no expression in tumors compared to healthy tissue, independent of *VHL* alteration. The 786-O cells showed increased HIF1A protein expression after 48 h under low oxygen and 10% FBS. EPO and erythropoietin receptor (EPOR) were significantly decreased in ccRCC without HIF1A expression. EPO mRNA increased in the 786-O cells at 3% O_2_ after 48 h, while the Caki-1 cells had low or no EPO expression. Hypoxia increased EPOR mRNA in the Caki-1 cells at 10% FBS, but decreased in the 786-O cells at 2% FBS after 48 h. JAK2/STAT5A activity was increased only in HIF1A-positive tumors. These results suggest that EPO/EPOR activation in ccRCC is mainly driven by low oxygen, not VHL regulation of hypoxia-related responses.

## 1. Introduction

Clear-cell renal cell carcinoma (ccRCC) is characterized by a pseudohypoxic intracellular state and genetic abnormalities, including mutation of the von Hippel–Lindau (VHL) tumor suppressor gene whose inactivation leads to stabilization of hypoxia-inducible factors (HIF1s) [1]. The erythropoietin (EPO) gene is under direct control of hypoxia through HIF1A binding to cis-acting DNA hypoxia-responsive elements of the EPO gene promoter [2]. EPO is required for erythropoiesis, but can also stimulate angiogenesis, tumor cell proliferation and survival in human renal cell carcinoma (RCC) [3,4,5]. In addition, RCC has been shown to express the EPO receptor (EPOR) and promote the proliferation of renal carcinoma cells in vitro [6]. EPO achieves its effect by binding to cell surface EPOR homodimers and activating Janus kinase 2 (JAK2) and downstream signal transducer and activator of transcription 5A (STAT5A) protein [7].

In ccRCC, EPO expression has been identified as a significant predictor of survival rates, with patients positive for EPO expression showing a better prognosis and survival, while those negative for EPO are twice as likely to die from RCC [8]. However, previous studies indicate that patients with tumors exhibiting high EPOR expression and elevated soluble EPO levels have significantly lower survival rates and extremely poor prognoses compared to other patients [9]. Moreover, the *VHL* mutations are crucial for RCC pathogenesis, as *VHL* inactivation is associated with the upregulation of hypoxia-inducible factors (HIF1 and HIF2), playing a more pronounced role in activating downstream pathways, including EPO production [10].

We analyzed the tumor and surrounding healthy tissue from patients with ccRCC after radical nephrectomy to explore hypoxia-related HIF1A and EPO expression and JAK2/STAT5 signaling pathways. We compared the human RCC 786-O cell line, a model with biallelic inactivation of *VHL*, and the human RCC Caki-1 cell line, a model with a wild type *VHL*, under normal and reduced oxygen levels. Furthermore, 786-O and Caki-1 cells were grown under conditions of reduced serum to demonstrate their influence on specific gene expression in both normal and low oxygen tension. Based on our results from the tumor and surrounding healthy tissue samples, HIF1A emerged as the primary determinant of *EPO*, *EPOR* and *JAK2/STAT5A* signaling pathway expression in ccRCC.

## 2. Results

### 2.1. Genetic Alteration in VHL

Preliminary characteristics of the tumor samples from the 43 ccRCC patients in the current study were reported previously [11]. Briefly, a single mutation of *VHL* was found in 16/43 (37.2%) tumors. The rest of the tumors (27 tumors) were analyzed with multiplex ligation-dependent probe amplification (MLPA), where intragenic deletions were found in 11 tumors. Altogether, genetic alteration in *VHL* was found in 27/43 (62.8%) of ccRCC samples. Genetic changes in the *VHL* were detected by loss of heterozygosity (LOH), whereby biallelic inactivation was detected in 23 samples and monoallelic inactivation in 4 samples. Hemizygosity was found in six samples. No genetic mismatch was detected in the remaining 10 samples.

### 2.2. HIF1A Expression in ccRCC

**Figure 1:**
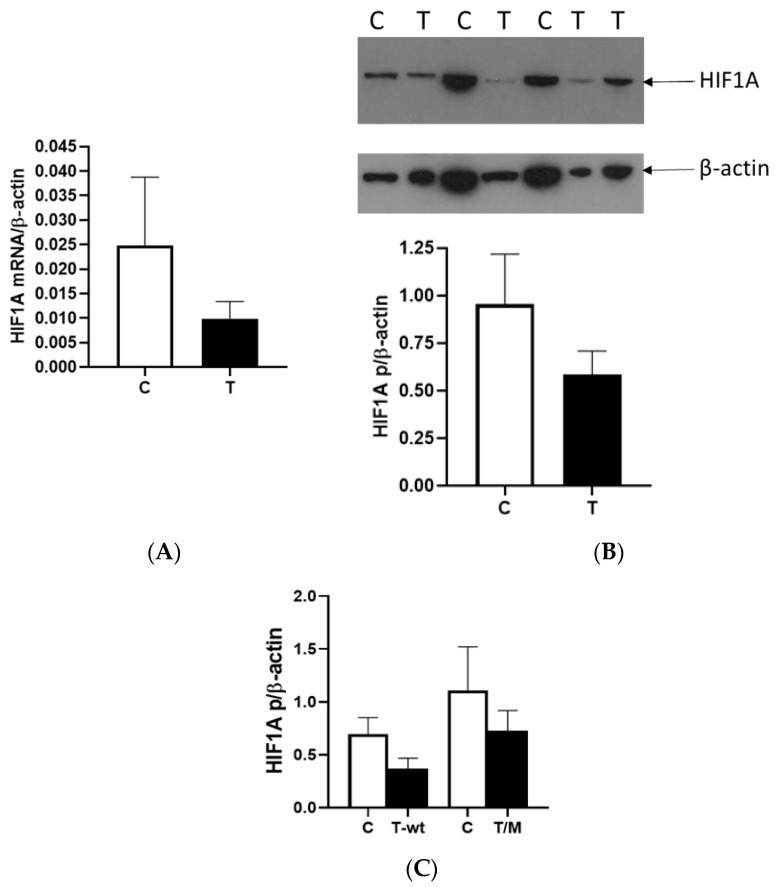
HIF1A expression in ccRCC. (**A**) HIF1A mRNA expression in ccRCC was determined by quantitative real-time RT-PCR in normal (C) and tumor (T) renal tissues. (**B**) Western blot and quantification of HIF1A (HIF1A p) in tumor cells (T) compared to control surrounding healthy tissue (C). (**C**) Quantification of HIF1A protein in tumor cells with wild-type *VHL* (T-wt) and mutated *VHL* (T/M) compared to control surrounding healthy tissue (C). Results were normalized to β-actin expression. Error bars represent SEM (*n* = 43).

The expression of HIF1A was examined by quantitative RT-PCR and Western blot analysis in ccRCC. HIF1A mRNA was decreased in tumor tissue compared to healthy tissue but without statistical significance (Figure 1A). HIF1A protein expression was almost analogous to mRNA expression and also without statistical significance (Figure 1B). Dividing the tumors into those that express the wild-type VHL gene and those with the mutated VHL gene, the quantification of HIF1A revealed decreased expression in both tumor types compared to the surrounding healthy tissue (Figure 1C). HIF1A had a tendency to be downregulated in tumor tissue independent of *VHL* alteration.

Among the 43 ccRCC samples, based on HIF1A protein expression, 27 tumors exhibited HIF1A expression and were categorized as positive (HIF1A+), while 16 tumors showed very low or no HIF1A expression and were classified as negative (HIF1A−).

### 2.3. HIF1A Expression in Caki-1 and 786-O Cell Lines

**Figure 2:**
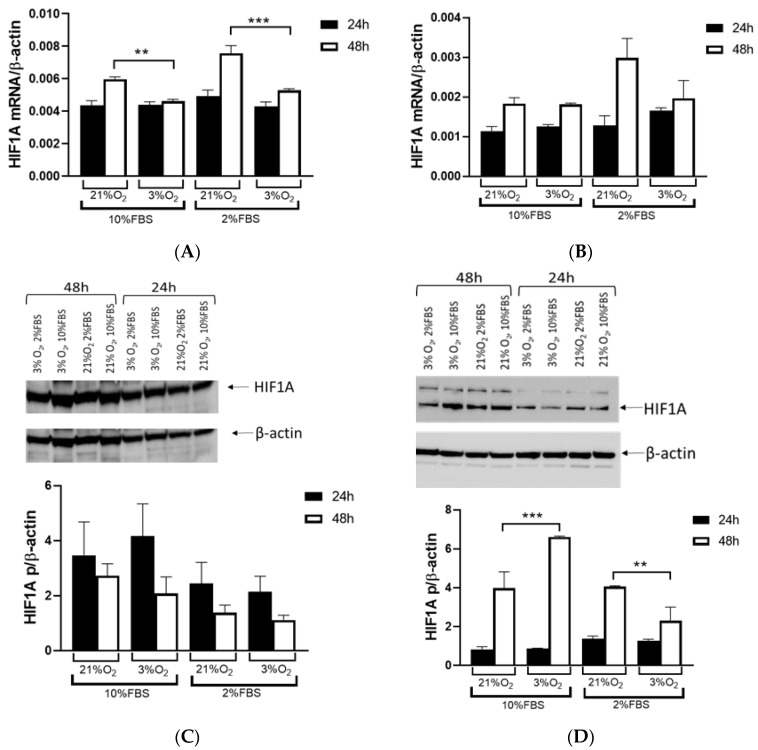
Quantitative real-time PCR and Western blot of HIF1A in Caki-1 and 786-O cells. (**A**) *HIF1A* expression was determined by quantitative real-time PCR in Caki-1 cells, with wild *VHL*, cultured in 10% fetal bovine serum (FBS) and 2% FBS at 21% and 3% oxygen for 24 (black bars) and 48 h (open bars). (**B**) Quantitative real-time PCR expression in 786-O cells with inactivated *VHL* cultured in 10% FBS and 2% FBS at 21% and 3% oxygen for 24 (black bars) and 48 h (open bars). (**C**) Western blot of HIF1A and quantification of HIF1A proteins in Caki-1 cells. (**D**) Western blot of HIF1A and quantification of HIF1A proteins in 786-O cells conducted by Western blotting. Results were normalized to β-actin expression. Error bars represent SEM (*n* = 3). ** *p* < 0.01, *** *p* < 0.001 vs. as indicated.

Regardless of VHL presence or absence, Caki-1 and 786-O cells expressed HIF1A under normal and low oxygen tension. To assess the effects of oxygen levels and serum influence as hormonal factors for cell proliferation and growth, the Caki-1 and 786-O cells were cultured in 2% and 10% fetal bovine serum (FBS) under 3% and 21% oxygen conditions. Caki-1 cells, with wild *VHL*, demonstrated lower expression of *HIF1A* at 3% oxygen after 48 h at 10% (*p* = 0.0088) and 2% FBS (*p* < 0.001, Figure 2A). There was no difference in the expression of mRNA HIF1A between normal and low oxygen tension in the 786-O cells with inactivated *VHL* after 24 and 48 h (Figure 2B). The pattern of HIF1A protein expression in the Caki-1 cells was similar to mRNA expression, and the quantification of HIF1A expression showed no differences regardless of oxygen levels or the percentage of FBS (Figure 2C). Quantification of Western blot analysis showed higher HIF1A protein expression in the 786-O cells after 48 h under low oxygen tension and 10% FBS (*p* < 0.001, Figure 2D). However, when cells were cultured in 2% serum, there was a decrease in HIF1A protein expression after 48 h, suggesting an important role of serum in HIF1A activity in the 786-O cells (*p* = 0.0026, Figure 2D).

### 2.4. Regulation of EPO Expression in ccRCC and Related Cell Lines

**Figure 3:**
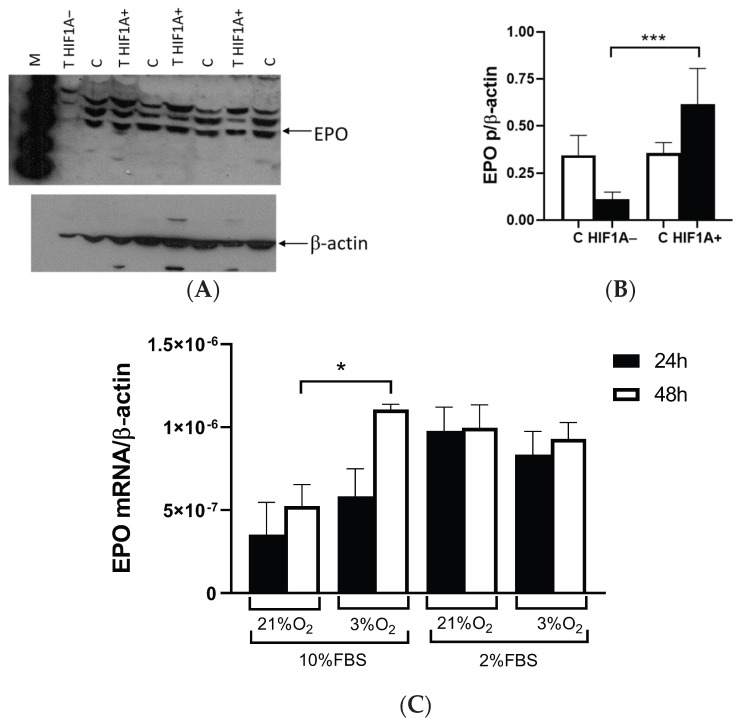
EPO expression in the ccRCC and 786-O cell line. (**A**) Western blot of EPO protein expression in HIF1A-negative (HIF1A−) and HIF1A-positive (HIF1A+) tumors and control surrounding healthy tissue (C). (**B**) Quantified EPO protein expression in HIF1A-negative (HIF1A−) and HIF1A-positive (HIF1A+) tumors and control surrounding healthy tissue (C). (**C**) *EPO* expression in 786-O cells with inactivated *VHL* at 21% and 3% oxygen for 24 (black bars) and 48 h (open bars). Results were normalized to β-actin expression. Error bars represent SEM (*n* = 3). * *p* < 0.05, *** *p* < 0.001 vs. as indicated.

Western blots HIF1A of ccRCC revealed that EPO protein expression was decreased in tumors without HIF1A compared to the control tissue (*p* = 0.0622, Figure 3A). Quantification of EPO protein in ccRCC showed that the loss of HIF1A protein was associated with very low EPO expression, while the HIF1A presence in the tumor indicated a strong upregulation of EPO compared to HIF1A-negative tumors (*p* < 0.001, Figure 3B). However, there was no statistical difference between control tissue and HIF1A-positive tumors. Also, no statistically significant differences in EPO mRNA expression levels were observed in the tumor and control tissues of the ccRCC samples (*p* = 0.8869). Further, two different cell lines revealed distinct *EPO* expression patterns; Caki-1 cells, with wild *VHL*, exhibited no or very weak expression of EPO mRNA (the Ct values were above 35 cycles), while 786-O cells with inactivated *VHL* had a tendency of increased *EPO* expression after 24 h in hypoxia, but without statistical significance. Significant EPO mRNA expression was observed at low oxygen tension in the 786-O cells after 48 h (*p* = 0.0136, Figure 3C). In the serum-depleted 786-0 samples grown under normal and hypoxic conditions, similar *EPO* expression was observed (Figure 3C), suggesting that the reduced serum concentration alone influences increased *EPO* expression independently of oxygen tension.

### 2.5. HIF1A Dependent Regulation of EPOR in ccRCC

**Figure 4:**
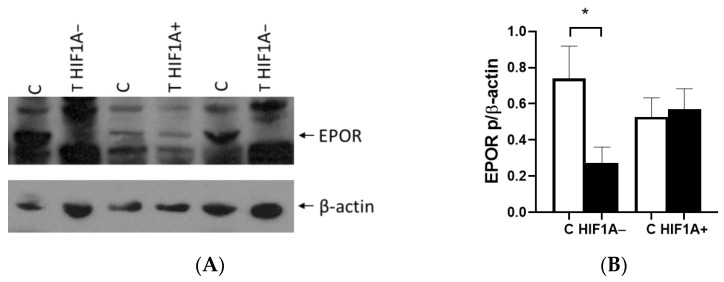
EPOR protein expression in ccRCC. (**A**) Western blot analysis of EPOR protein in surrounding healthy tissue (C) compared to HIF1A-negative (HIF1A−) and HIF1A-positive (HIF1A+) ccRCC tissues. (**B**) Quantified Western blot analysis of EPOR protein in HIF1A-negative (HIF1A−) and HIF1A-positive (HIF1A+) ccRCC tissues and in surrounding healthy tissue (C). Results were normalized to β-actin expression. Error bars represent SEM. * *p* < 0.05.

EPO acts by binding to its receptor EPOR, which is expressed in ccRCC and surrounding normal cells. Western blot showed EPOR protein expression in all examined samples (Figure 4A). Unlike EPO, the EPOR expression in HIF1A protein-positive tumors was similar between tumors and control tissues. The lack of HIF1A protein decreased the expression of EPOR in tumor tissue (*p* = 0.0246, Figure 4B). When HIF1A was present in the tumor tissue, the EPOR expression was inducted compared to the HIF1A-negative tumors and the level was similar to that of the surrounding normal tissue (Figure 4B).

### 2.6. Regulation of EPOR in Caki-1 and 786-O Cells

**Figure 5:**
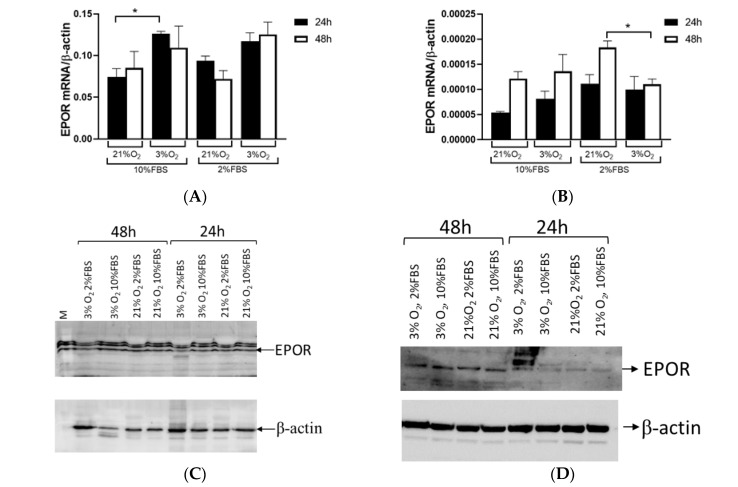

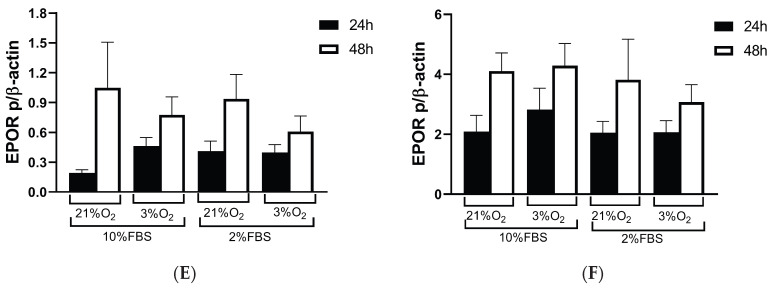
EPOR expressions in Caki-1 and 786-O cells cultured at 21% and 3% oxygen for 24 (black bars) and 48 h (open bars) with 10% and 2% fetal bovine serum (FBS). (**A**) RT-PCR of *EPOR* in Caki-1 cells, with wild *VHL*. (**B**) RT-PCR of *EPOR* in 786-O cells, with inactivated *VHL*. (**C**) Western blotting of EPOR in Caki-1 cells. (**D**) Western blotting of EPOR in 786-O cells. (**E**) Quantified Western blotting of EPOR in Caki-1 cells. (**F**) Quantified Western blotting of EPOR in 786-O cells. Results were normalized to β-actin expression. Error bars represent SEM (*n* = 3). * *p* < 0.05 compared normoxia to hypoxia.

EPOR was detected at the mRNA and protein levels in the Caki-1 and 786-O cells. In the Caki-1 cells, with wild *VHL*, hypoxia significantly enhanced EPOR mRNA synthesis, particularly after 24 h at 10% FBS (*p* = 0.0454, Figure 5A), while serum deprivation also showed an increase, which was not statistically significant. Conversely, the 786-O cells with inactivated *VHL* exhibited an increase in EPOR mRNA after 48 h at 10% FBS, but low serum and hypoxic conditions led to reduced expression (*p* = 0.016, Figure 5B). The Western blot data correlated with the EPOR mRNA results (Figure 5C–F). Quantification of the EPOR protein expression in the Caki-1 cells showed similar expression to mRNA levels after 24 h at 10% FBS (*p* = 0.072, Figure 5E). The comparisons of protein expression yielded no statistically significant differences in the 786-O cells under different oxygen and serum conditions, while the protein EPOR expression was generally higher after 48 h (Figure 5F).

### 2.7. JAK2/STAT5A Signaling in ccRCC

**Figure 6:**
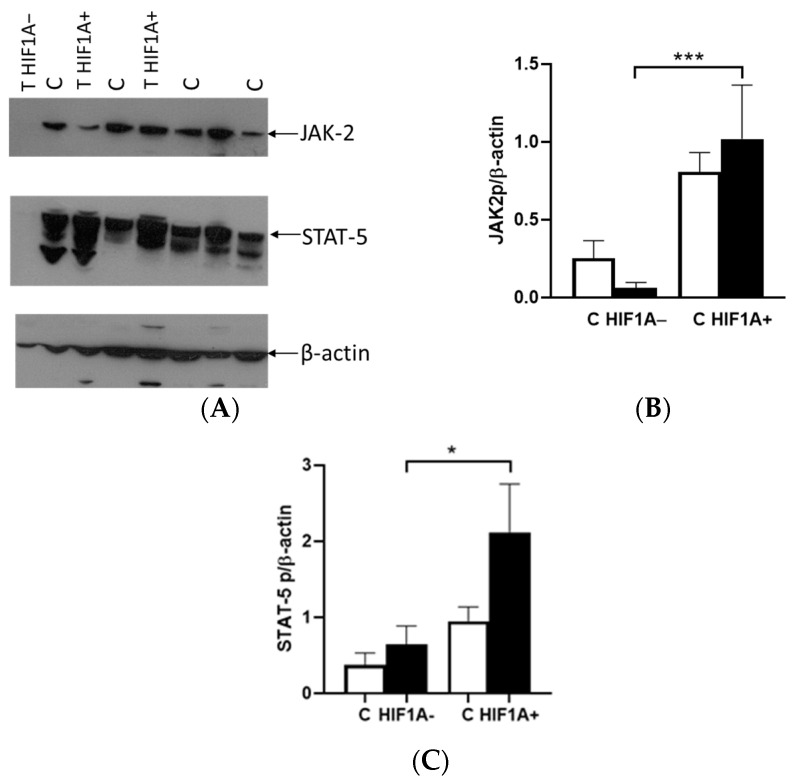
Western blot analysis of JAK2 and STAT5A signaling in ccRCC. (**A**) Western blot of JAK2, STAT5A and β-actin. (**B**) Quantitative expression of JAK2 protein in HIF1A-negative (HIF1A−) and HIF1A-positive (HIF1A+) tumors and control surrounding healthy tissue (C). (**C**) Quantitative expression of STAT5A protein in HIF1A-negative (HIF1A−) and HIF1A-positive (HIF1A+) tumors and control surrounding healthy tissue (C). Results were normalized to β-actin expression. Error bars represent SEM. * *p* < 0.05, *** *p* < 0.001 vs. as indicated.

The binding of EPO to EPOR activates downstream pathways such as JAK2/STAT5A [7]. Western blotting performed in ccRCC and healthy surrounding tissues showed similar expression of JAK2 and STAT5A signaling. However, when tumor cells are divided in tumor tissue with and without HIF1A expression, statistical significance was observed in JAK2 expression (Figure 6A). The quantification of JAK2 in the HIF1A-negative tumor showed the low expression of JAK2 protein compared to control tissue; however, in the HIF1A-positive tumor, the strong expression of JAK2 was observed compared to HIF1A-negative tumor (*p* < 0.001, Figure 6B). There was a tendency for greater STAT5A protein expression in tumor tissue compared to surrounding healthy tissue and without statistical significance. In the tumors divided as HIF1A-positive and HIF1A-negative, statistical significance in terms of expression was observed particularly in the HIF1A-positive tumor (*p* = 0.041, Figure 6C). 

## 3. Discussion

RCC is one of the most common types of cancer, characterized by high aggressiveness, invasiveness and metastatic potential, features that lead to poor prognosis and high mortality rate [12]. A common event in ccRCC is inactivation of the VHL gene product, pVHL and deregulation of hypoxia signaling pathways. Germline mutations in pVHL are responsible for ccRCC, followed by the disruption of HIF1A degradation [13]. However, it remains unsolved whether impaired HIF1A degradation without *VHL* mutations is sufficient to cause renal tumors or if they must occur in combination with *VHL* mutations. We analyzed the tumor samples for *VHL* mutation where a genetic alteration in the *VHL* was in more than 75% of the ccRCC samples either by allelic deletion or mutations and the rest of the tumors were without genetic mismatch in the VHL gene. These results are consistent with results published previously, where *VHL* was shown to be affected in more than 80% of ccRCC cases [14].

Moreover, we used two cell lines, 786-O (which has biallelic *VHL* mutations) and Caki-1 (which lacks *VHL* mutations), cultured under two different serum conditions: normal serum (10% FBS) and low serum (2% FBS), to examine how variations in cell nutrition affect the expression of hypoxia-related genes. Inadequate nutrient intake leads to oxidative stress, disrupting cellular homeostasis and activating signaling pathways that enable cancer cells to adapt and survive. However, the mechanisms facilitating this adaptation and survival remain unclear. In prostate cancer, nutrient deprivation enhances cellular adaptability to oxidative stress and/or improves survival mechanisms against anti-tumor agents [15].

We observed a reduction in HIF1A expression within tumor tissue compared to surrounding tissue at the mRNA and protein levels, with a third of all tumor RCC samples showing very low or no HIF1A expression. Different studies reported contrasting patterns of HIF1 expression within cells, and recent studies showed that HIF1A and HIF3A were more expressed in the nucleus than in the cytoplasm, particularly in ccRCC compared to other types of RCC [16]. In our study, we isolated the total proteins from the tumor and healthy tissue samples to investigate the factors responsible for the lower expression of HIF1A without separating proteins from the nucleus and cytoplasm. Second, many studies suggested that HIF1A acts as a tumor suppressor, so its reduced activity in relation to surrounding healthy tissue could contribute to the initiation of tumor development in the kidney, a finding consistent with our data [17]. When we used cell lines with (786-O) and without (Caki-1) *VHL* mutations and exposed them to normal and hypoxic conditions for 24 and 48 h, we observed that the Caki-1 cells exhibited up to 60% lower HIF1A expression under reduced oxygen levels only after 48 h at the mRNA level, regardless of serum concentration, while the protein levels showed no difference. In contrast, when we repeated the same conditions with the 786-O cells, HIF1A expression was significantly higher, increased by 60% after 48 h, particularly under hypoxic conditions and 10% serum. However, reducing both serum concentration and oxygen levels decreased HIF1A expression 2-fold in the 786-O cells with biallelic inactivation of *VHL*. This result suggested that the initial mutation of *VHL* in tumor tissue enhanced *HIF1A* activity, indicating that adequate nutrition is essential for its full activity, supporting tumor growth. Further, while HIF1A acts as a tumor suppressor, HIF2A promotes oncogenic potential by driving tumor progression and metastasis through activation of hypoxia-sensitive signaling pathways and overexpression of HIF2A target genes [18]. 

Additionally, HIF2A plays a critical role in RCC progression by enhancing oncogenic potential, driving tumor progression and metastasis through the activation of hypoxia-sensitive signaling pathways and the overexpression of its target genes [16,19]. Additionally, HIF1A is not only an important regulator in the acute response to hypoxia, enabling cells to meet energy demands when oxygen is limited, but it also regulates genes involved in glycolysis, facilitating anaerobic metabolism. There is likely a switch to HIF2A, which is more tissue-specific and primarily found in endothelial cells, interstitial fibroblasts and certain types of cancer cells. In these contexts, HIF2A becomes more prominent in chronic hypoxia adaptation and plays a crucial role in the progression of hypoxia-driven cancers, such as RCC [20].

EPO, a hypoxia-inducible gene, is produced by peritubular interstitial fibroblasts located in the renal cortex, particularly in the juxtamedullary region and outer medulla [2]. We demonstrated the critical role of the HIF1A gene in EPO expression using samples from patients with ccRCC. Our findings revealed that tumors expressing EPO could be categorized into HIF1A-expressing and non-expressing groups alongside healthy control tissue, highlighting the significant influence of HIF1A on EPO regulation. We found that EPO expression was fivefold lower in ccRCC lacking HIF1A, while HIF1A-positive tumors exhibited a statistically significant increase in EPO expression. However, we found no difference in EPO expression between tumor and healthy tissue, nor in cells with *VHL* allelic alterations. Since Caki-1 is a cell line with epithelial morphology isolated from kidney tissues, it is likely that they do not express *EPO*, which may explain the lack of expression observed in our study. However, there was *EPO* expression with a tendency to increase in the first 24 h, achieving statistical significance and a twofold increase after 48 h under low oxygen conditions in the 786-O cells with 10% serum. Interestingly, when serum was reduced, reflecting decreased cell nutrition under both normal and hypoxic conditions, *EPO* expression remained very similar but still higher compared to 10% FBS. This suggests that mutation in *VHL* leads to the initial activation of EPO expression in renal tumors, subsequently enhancing HIF1A activity, and sustained hypoxia is essential for maintaining EPO expression in the tumor microenvironment.

EPOR expression showed no significant difference between tumor and healthy tissue in ccRCC. However, when tumors were categorized based on HIF1A expression, the results were almost consistent with EPO expression. EPOR levels in tumor tissue were reduced compared to surrounding healthy tissue in the absence of HIF1A. EPOR expression remains comparable to that in healthy tissue if HIF1A is present. We assessed *EPOR* expression in the Caki-1 cells and found no significant differences in expression over time (24 vs. 48 h), but there was a notable change in expression between different oxygen concentrations (21% vs. 3% O_2_) of about 70% at both time points. A low serum concentration did not affect *EPOR* expression in the Caki-1 cells. In contrast, *EPOR* expression varied based on culture duration rather than oxygen levels in the 786-O cells, indicating that *EPOR* expression was higher in response to reduced oxygen in tumor cells, while in tumor cells with a mutation in *VHL*, it depended on the length of cultivation. When 786-O cells were exposed to low serum and low oxygen, *EPOR* expression decreased by 70%, suggesting that not only HIF1A but also serum played an important role in regulating *EPOR* expression. This confirms that the early stages of tumor development and later stages of tumor growth were differentially regulated and characterized by different gene expression profiles and regulatory mechanisms. Further, tumors with HIF1A activity displayed normal EPOR expression and a tendency for increased EPO secretion, whereas HIF1A-negative tumors exhibit reduced levels of both EPO and EPOR compared to surrounding healthy tissue. The current results were consistent with a previous report of ccRCC that linked EPO expression to the TMN stage and nuclear grades in ccRCC samples, while strong significance was observed in the early-stage group (T1G1), suggesting that EPO overexpression in the early stages of the tumor may play a crucial role in promoting cell proliferation [6].

The binding of EPO to EPOR induces the receptor’s conformational change, resulting in the autophosphorylation of JAK2 kinases, which serve as docking sites for a variety of intracellular pathway activators and STATs, such as STAT5A. The difference in the expression between the tumor and healthy tissues was not observed in relation to the presence or absence of HIF1A protein. In cases where HIF1A is expressed, there was an upregulation of JAK2 and STAT5A proteins in tumor tissue, alongside EPOR. However, in tumors lacking HIF1A expression, the levels of these proteins were significantly reduced compared to HIF1A-expressing tumors. Interestingly, the expression of these proteins was also detected in healthy tissue, indicating that the tumor microenvironment and HIF1A status play a crucial role in modulating their expression. The classification of tumors based on *VHL* did not reveal any significant differences in the expression levels of either JAK2 or STAT5A proteins. This suggests that the expression of these proteins may be independent of *VHL*, in contrast to the important role of HIF1A and microenvironment progression.

All these results suggest that the use of cell culture models may not effectively replicate the complex processes occurring in tumors, as they may only be suitable for studying early tumor development. Initial changes likely involve mutated *VHL*, but as the tumor progresses, VHL status may no longer serve as a key trigger for tumor growth and metastasis. This indicates that more sophisticated models, perhaps incorporating the tumor microenvironment and later-stage alterations, are necessary for a comprehensive understanding of tumor biology and progression. While the expression differences between tumor and healthy tissue were not observed, they were notably influenced by the presence or absence of HIF1A. Interestingly, these proteins were also expressed in healthy tissue, highlighting the critical roles of the tumor microenvironment and HIF1A status in regulating their expression. This underscores the potential for targeting these pathways in therapeutic strategies.

## 4. Material and Methods

### 4.1. Tumor Tissue Samples

All ccRCC patients were diagnosed at the Clinic of Urology, University Clinical Center of Serbia, Belgrade, Serbia. We included 43 histologically confirmed patients (30 men and 13 women, with an average age of 57.6 ± 11.7) with ccRCC who underwent surgery. This study was performed following the Declaration of Helsinki after approval by the Ethics Committee of the University Clinical Center of Serbia (protocol code 476/7, approved 2007). Written informed consent was obtained from all participants. Samples from the tumor and surrounding healthy tissue were collected from 43 patients scheduled for the surgical removal of a kidney. Immediately after radical nephrectomy, a pathologist excised samples from the tumor and surrounding healthy tissue area, which were immediately stored as well as immersed in RNAlater stabilizing solution (RNAlater RNA Stabilization Reagent, Qiagen, Hilden, Germany) at −80 °C until used.

### 4.2. Cell Culture

Human RCC cell lines 786-O (with biallelic inactivation of *VHL*) and Caki-1 (with a wild type VHL) were obtained from the American Type Culture Collection (ATCC, Manassas, VA, USA). The 786-O cells were grown in RPMI 1640 containing 4500 mg/L glucose, 2 mM L-glutamine, 10 mM HEPES, 1 mM sodium pyruvate, 1500 mg/L sodium bicarbonate (ATCC) and supplemented with 10% FBS (Biowest, Nuaille, France), penicillin and streptomycin (P/S, Penicillin-Streptomycin, Biowest). The 786-O cells were washed with PBS and seeded in plates up to 48 h with medium containing a reduced amount of FBS (2%). The Caki-1 cells were cultured in McCoy’s medium (McCoy’s 5A, Biowest) containing 10% FBS and P/S. All cells were maintained at 5% CO_2_ and balanced 95% room air in a humidified 37 °C incubator (Forma Scientific, Marietta, OH, USA). For experiments, the Caki-1 cells were washed with PBS and transferred to a medium containing 2% and 10% FBS. An incubator (Proox Culture Chamber with O_2_ and CO_2_ regulators BioSpherix, Ltd., Redfield, NY, USA) with low oxygen was used for 3% oxygen tension.

### 4.3. Isolation of DNA in Tissue

Isolation of DNA from tissue and blood samples has been described previously [11]. Briefly, DNA was isolated from 100 mg of the tumor and surrounding tissues with extraction buffer (100 mM NaCl, 10 mM Tris-Cl, 25 mM EDTA), SDS (Sodium dodecyl sulfate) and proteinase-K. TE buffer (10 mM Tris and 1 mM EDTA) was added for the isolation of DNA from peripheral blood. Then, phenol-chloroform-isoamyl alcohol (25:24:1) was used for extraction, and ammonium acetate and cold absolute ethanol were used for precipitation. The pellet was washed with 70% ethanol and diluted in water for sequencing.

### 4.4. DNA Sequencing in Tissue

DNA sequencing from tissue and blood samples was previously described [11]. Briefly, genomic DNA obtained from tissue (tumor and surrounding healthy tissue) and blood were used for the PCR reaction (Fermentas, Hamburg, Germany). PCR products for the VHL gene were purified (Quiquick, Quiagen, Hilden, Germany) and ready for sequencing on ABI PRISM 3130 Genetic Analyzer (Applied Biosystems, Waltham, MA, USA) [11]. A Big Dye Terminator v3.1 and a v1.1 Ready Reaction Cycle Sequencing Kit (Applied Biosystems) were used for the PCR reaction. ABI DNA Sequencing Analysis Software v5.2 (Applied Biosystems) was used to analyze data.

### 4.5. Multiplex Ligation-Dependent Probe Amplification (MLPA) Analysis

MLPA analysis was previously described [11]. Briefly, prepared DNA samples were used for MLPA analysis according to the protocol from SALSA MLPA P016-C2 VHL kit (MRC-Holland, Amsterdam, The Netherlands). DNA samples obtained from healthy controls were also included. After ligation and denaturation, PCR products were analyzed with an ABI PRISM 3130 Genetic Analyzer (Applied Biosystems) with LIZ-500-labeled internal size standard. Data were obtained according to GeneMapper Software (GeneMapper Software, Version 3.7, Applied Biosystems). Coffalyser Net Software was used for DNA fragment analysis and comparative analysis of samples (Coffalyser.Net MLPA Data Analysis, v.140721.1958, MRC-Holland).

### 4.6. Loss of Heterozygosity (LOH) Analysis in Tissue

LOH analysis was performed as previously described [11]. Briefly, six polymorphic microsatellite markers for the VHL gene (D3S1038, D3S1435, D3S1317, D3S1597, D3S1537 and D3S3691) were used in a single PCR reaction and electrophoretically separated using ABI PRISM 3130 Genetic Analyzer (Applied Biosystems). PCR products were sized in comparison to the LIZ-500-labeled internal size standard. GeneMapper Software (Version 3.7, Applied Biosystems) was used for data analysis.

### 4.7. RNA Isolation and Quantitative Real-Time Polymerase Chain Reaction

786-O and Caki-1 cells were cultured at various time points, harvested after the incubation period, washed with PBS, trypsinized and then prepared for RNA isolation. In addition to cells, tumor and surrounding healthy tissues weighing up to 30 mg were disrupted with mortar and pestles and further used for RNA isolation. Cells and tissues were homogenized using QIAshredder homogenizer (QIAshredder, Qiagen). The QIAshredder spin columns were placed in a 2 mL collection tube and spun for 2 min at maximum speed in a microcentrifuge. Total RNA was isolated using an RNA isolation kit (RNAeasy Mini Kit, Qiagen) according to the manufacturer’s protocol. The tissue samples were treated with DNase I, RNase-free (DNase I, RNase-free, Thermo Fisher Scientific, Waltham, MA, USA). RNA from each sample was used for first-strand cDNA synthesis using M-MuLV reverse transcriptase and oligo d(T)_18_ (Thermo Fisher Scientific). Quantitative real-time RT–polymerase chain reaction (PCR) analyses were performed using a LightCycler 1.5 (Rohe Diagnostics GmhH, Mannheim, Germany), LightCycler 480 (Rohe Diagnostics GmhH) and an ABI PRISM 3130 Genetic Analyzer (Applied Biosystems) with specific probes and primers (TIB MOLBIOL GmbH, Berlin, Germany) as previously described [21]. β-Actin was used as an internal control for the total amount of RNA analyzed.

### 4.8. Protein Analyses of Tissue and Cell Lines

The 786-O and Caki-1 cells were washed twice with cold PBS, treated with lysis RIPA buffer and protease inhibitors (Complete Protease Inhibitor Cocktail Tablets, Roche Diagnostics GmhH, Penzberg, Germany) and scraped from the plate. The cell lysate was centrifuged for 10 min and the supernatant was transferred to a new tube for further analysis. Tissue samples were weighed up to 100 mg, disrupted with a mortar and pestle, lysed in RIPA buffer with protease inhibitors and left on ice for 30 min. The pellet was sonicated three times for 10 s with a pause of 1 min and then kept on ice for 30 min. Then, the mixture was centrifuged at 11,000 rpm for 20 min to pellet the cell debris. The proteins in all samples were separated by sodium dodecyl sulfate–polyacrylamide gel electrophoresis (SDS-PAGE, Thermo Fisher Scientific). After electrophoresis, proteins were transferred to a nitrocellulose membrane. The membrane was then blocked with 5% milk with 0.1% Tween 20 for 1 h at room temperature. The membrane was probed with different antibodies, HIF1A (BD Transduction Laboratories, Franklin Lakes, NJ, USA), EPO (Santa Cruz Biotechnology, Inc., Santa Cruz, CA, USA), EPOR (Santa Cruz Biotechnology, Inc.), JAK2 (Cell Signaling Technology, Danvers, MA, USA) and STAT5A (Cell Signaling Technology) overnight at 4 °C. For the secondary antibody, horseradish peroxide-labeled immunoglobulin G (IgG) was used. Hyperfilm was used to visualize the secondary antibody by the enhanced chemiluminescence (ECL; Amersham Pharmacia Biotech, Piscataway, NJ, USA). The membrane was then stripped for one hour at 55 °C, washed, blocked with milk and incubated with the β-actin antibody (Anti-beta actin antibody, Abcam, Cambridge, UK) overnight at 4 °C and then visualized. Quantification of proteins was performed with the software Image LabTM Version 6.0.0 build 25 (Biorad Laboratories, Inc., Hercules, CA, USA).

### 4.9. Statistical Analysis

Data calculations were performed using SPSS, Inc. (Chicago, IL, USA) for Windows, version 13.0. The results are presented as mean ± standard error of the mean. Depending on data distribution, analysis of variance (ANOVA) with Bonferonni’s correction for post hoc multiple group comparisons or the Kruskal–Wallis nonparametric ANOVA followed by the Mann–Whitney test was used to detect differences between tumorous and corresponding normal tissues. A signal ratio between pairs of tumorous and corresponding healthy tissues was calculated for each of the analyzed proteins.

## 5. Conclusions

According to our data, short-term exposure of tumor cells to low oxygen and adequate nutrition is crucial for full HIF1A activation, which supports tumor growth in the beginning. In contrast, under chronic hypoxia, HIF1A expression appears to decrease over time. This suggests that pseudohypoxia, along with the tumor microenvironment and the effects of HIF1A activation, leads to increased expression of hypoxia-related genes such as *EPO*. However, not all tumors express HIF1A. In tumors expressing HIF1A, elevated EPO expression contributes to tumor progression by stimulating EPOR and activating downstream signaling pathways, such as JAK2-STAT5. It is also likely that other hypoxia-related genes and signaling pathways further contribute to the growth and metastasis of ccRCC.

## Data Availability

The data are not publicly available due to privacy and ethical restrictions.
